# The patient’s decision dilemma after screening coronary computed tomography angiography—adult-type ALCAPA with a multimodality imaging approach: a case report

**DOI:** 10.1093/ehjcr/ytaf511

**Published:** 2025-10-09

**Authors:** Anna Jöbstl, Lukas Wirth, Gudrun M Feuchtner, Johannes Mair, Gerlig Widmann

**Affiliations:** Department of Radiology, Medical University of Innsbruck, Anichstraße 35, 6020 Innsbruck, Tirol, Austria; Department of Nuclear Medicine, Medical University of Innsbruck, Anichstraße 35, 6020 Innsbruck, Tirol, Austria; Department of Radiology, Medical University of Innsbruck, Anichstraße 35, 6020 Innsbruck, Tirol, Austria; Department of Internal Medicine III—Cardiology and Angiology, Medical University of Innsbruck, Anichstraße 35, 6020 Innsbruck, Tirol, Austria; Department of Radiology, Medical University of Innsbruck, Anichstraße 35, 6020 Innsbruck, Tirol, Austria

**Keywords:** Congenital heart disease, Multimodality imaging, ALCAPA, Adult-type ALCAPA, Case report

## Abstract

**Background:**

Anomalous left coronary artery from the pulmonary artery (ALCAPA) is a very rare, congenital condition. Patients typically exhibit symptoms within the first few weeks of life. ‘Adult-type ALCAPA’, which presents later in life, is even rarer and is mostly detected due to symptoms of heart failure or after life-threatening arrhythmias.

**Case summary:**

We present the case of a 53-year-old asymptomatic male, who was incidentally diagnosed with adult-type ALCAPA during a coronary computed tomography angiography (CCTA) screening performed for cardiac risk stratification. Further cardiac investigations included echocardiography, myocardial perfusion scintigraphy, invasive coronary angiography (ICA), and cardiac magnetic resonance imaging (CMRI). As he had never experienced any cardiac symptoms at rest or during exercise throughout his life, the patient has not yet decided to undergo the recommended cardiac surgery. The patient is currently seeking a second opinion.

**Discussion:**

Anomalous left coronary artery from the pulmonary artery is rare, but the increased use of CCTA for risk stratification in asymptomatic patients might lead to more frequent diagnosis in older individuals. As with our patient, a dilemma may arise when deciding on its treatment, because the guideline-recommended treatment is surgical correction in all patients. However, there is still little evidence to support this recommendation for older, asymptomatic patients, making advice and decisions difficult for such individuals.

Learning points

- Anomalous left coronary artery from pulmonary artery (ALCAPA) is a rare congenital disorder with only 10%–15% of all untreated patients living to adulthood. Recommended treatment is surgical to avoid sudden cardiac arrest/death; at the moment, there are no recommendations for medical treatment.
- Because of increasing use of (screening) cardiac computed tomography (CT) angiography incidence of ALCAPA in adult and maybe asymptomatic patients might rise in the future and recommendations for medical treatment might get necessary.

## Primary specialties involved other than cardiology

Radiology and nuclear medicine.

## Introduction

Anomalous left coronary artery from the pulmonary artery (ALCAPA), also known as the Bland–White–Garland syndrome, is a very rare, congenital, mostly isolated disorder, with an estimated incidence of 1 in 300 000 live births.^1^ Two large studies found ALCAPA present on computed tomography (CT) in 0.043% of cases and on invasive coronary angiography (ICA) in 0.008% of cases, respectively.^2^ Immediately after birth, all patients with ALCAPA are asymptomatic due to a still-open ductus arteriosus. The ductus arteriosus normally closes within 2–3 weeks after birth, resulting in a gradual decrease in pulmonary pressure compared to systemic and, in particular, coronary pressure.^3^ Consequently, the left coronary artery (LCA) draws blood via physiological anastomoses from the right coronary artery (RCA). The onset and severity of symptoms depend on the promptness and extent of the development of collateralization, as well as on additional systemic collateral blood flow to the LCA. It is thought that a predominant RCA and a narrow LCA ostium are thought to support long-term survival.^4,5^

In ∼90% of cases, collateralization is insufficient, resulting in the subsequent development of heart failure symptoms, such as dyspnoea, tachypnoea, difficulty feeding, fatigue and failure to thrive, and, in most severe cases, myocardial infarction and sudden cardiac death (SCD) within the first 8 weeks of life (infant type).^3^ However, if the above-mentioned supporting features are present, patients can survive into adulthood (adult-type ALCAPA) without developing symptoms and may even remain asymptomatic for decades.^6^

Yau *et al*.^6^ reviewed 151 cases of adult-type ALCAPA and categorized the clinical presentation as ‘asymptomatic’, ‘subacute’ (with angina, dyspnoea, palpitations, or fatigue), or ‘life-threatening’ (with ventricular arrhythmias, syncope, or SCD-survival or SCD). The average age at diagnosis was 41 years, with 103 patients younger than and 48 patients older than 50 years.^6^ Females were affected more frequently. Only 14% of patients were asymptomatic. Life-threatening presentations were markedly more frequent in younger patients (22% vs. 8%), while older patients more frequently presented with subacute symptoms (75% vs. 65%) or no symptoms (17% vs. 13%).^6^ The average age at presentation of the life-threatening form was 33 years, affecting 18% of all adult-type ALCAPA patients. Seven percent of all patients died at an average age of 31 years.^6^

According to the European and American guidelines, surgical repair is recommended for all patients with ALCAPA (Class 1C recommendation), regardless of age or symptoms,^3,7^ due to the risk of ischaemia-triggered SCD.^3^ All surgical approaches aim to restore a dual coronary system for an arterial blood supply of the whole heart.^3^ The method of choice is direct reimplantation of the coronary artery into the aorta; alternatively, transpulmonary baffling (the Takeuchi procedure) is rarely used nowadays.^8^ Aortocoronary bypass grafting with closure of the LCA origin is mainly recommended for adults with significant concomitant CAD to ensure the long-term patency of the grafts.^3,4,7–10^ A significant myocardial scar (>50% of the myocardial thickness) on cardiac magnetic resonance imaging (CMRI) is considered as an indication for the secondary preventive implantation of an additional ICD.^11^

## Summary figure

**Figure:**
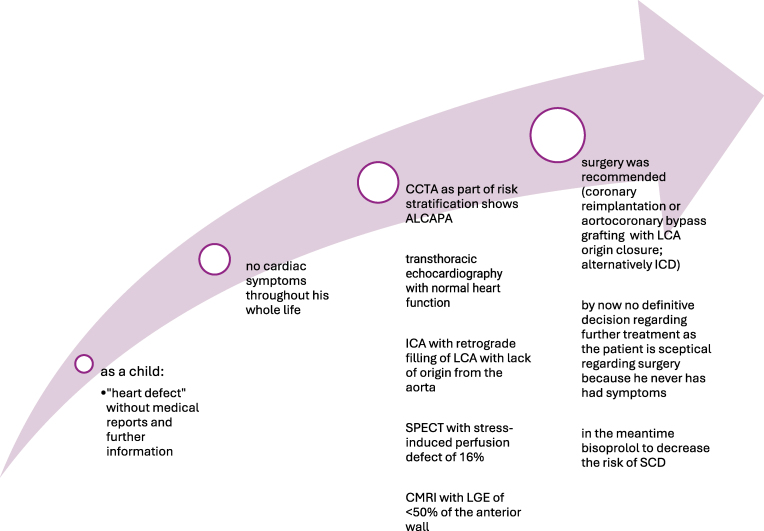


## Timeline

**Figure:**
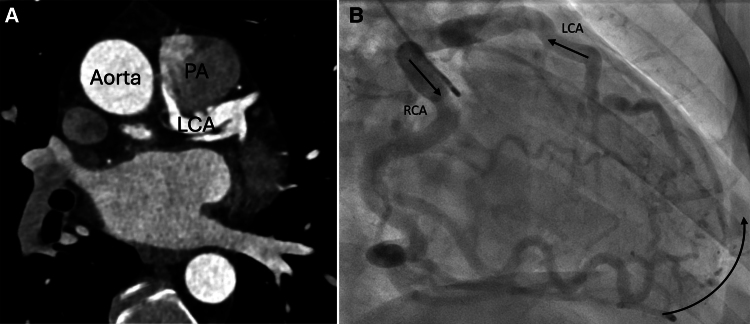
Adult-type ALCAPA: (*A*) CCTA shows the anomalous origin of the LCA from the PA instead of from the aorta. Contrast spillover from the LCA into the PA, as a pathophysiologic consequence of this variant. (*B*) ICA with contrast injection into the RCA shows pronounced collaterals and contrast spillover into the LCA (arrows). ALCAPA, anomalous left coronary artery from pulmonary artery; CCTA, coronary computed tomography angiography; LCA, left coronary artery; PA, pulmonary artery; RCA, right coronary artery; ICA, invasive coronary angiography.

## Case summary

A 53-year-old, male, white, Austrian patient was referred for CCTA as part of risk stratification during a routine preventive medical examination. Throughout his whole life, the patient had denied experiencing any cardiac symptoms, even under heavy exertion. He had a body mass index of 28.7, indicating mild obesity, and smoked five cigarettes a day. His cardiopulmonary physical examination and exercise capacity were normal. His previous medical history revealed that he had been diagnosed with a ‘heart defect’ as a child (no medical reports were available anymore). There was no family history of cardiac disease.

**Figure 1:**
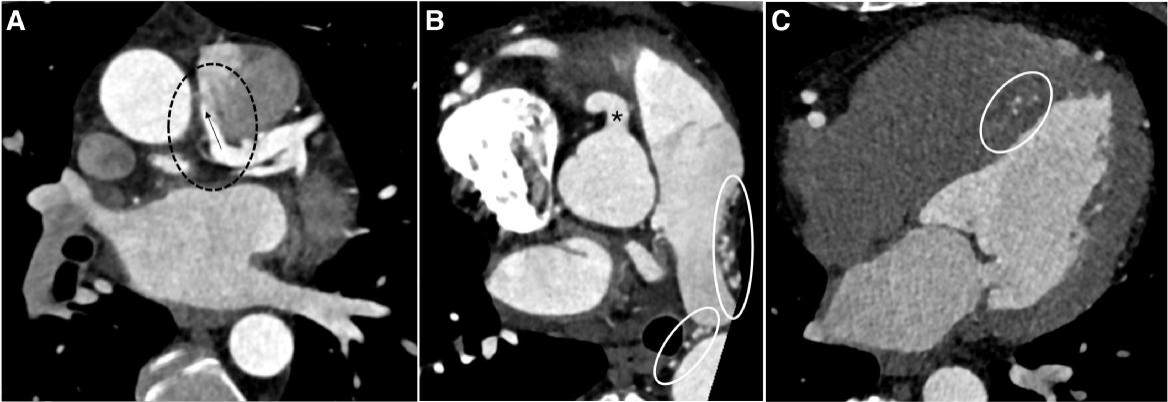
Coronary computed tomography angiography (CCTA). (*A*) Anomalous origin of the left coronary artery from the posteromedial side of the pulmonary trunk (black dashed circle) with contrast spillover into the pulmonary artery (black arrow). (*B*) Correct position of the origin of the right coronary artery (asterisk). Note multiple mediastinal collaterals (white circles) fed by bronchial arteries. (*C*) Prominent septal collaterals (white circle) fed by the right coronary artery.

**Figure 2:**
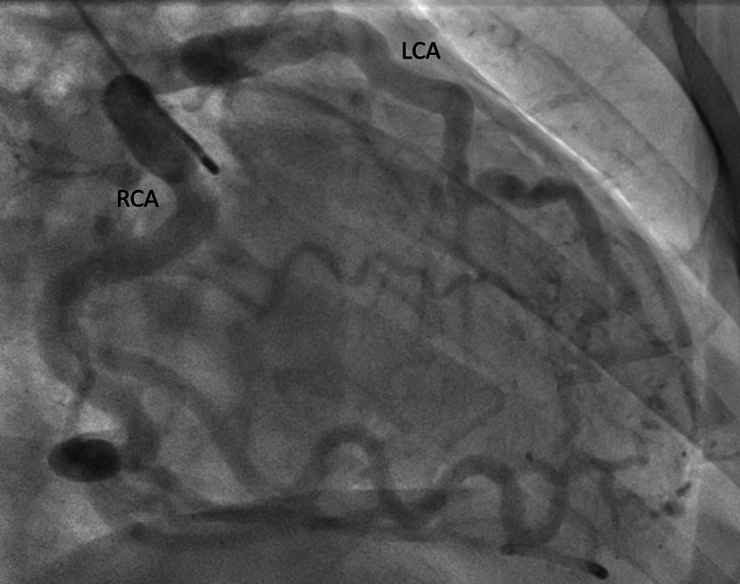
Invasive coronary angiography (ICA). Selective right coronary contrast injection shows a dilated right coronary artery (RCA) with retrograde perfusion of the left coronary artery (LCA) up to its anomalous origin in the pulmonary artery through multiple collateral pathways (please also refer to *Movie 1*).

Coronary computed tomography angiography revealed that the LCA originated from the pulmonary trunk (*Figure 1A*). There were collateral vessels including septal collaterals (*Figure 1B* and *C*) from the RCA resulting in retrograde perfusion of the LCA and contrast agent spillover into the pulmonary trunk (*Figure 1A*). Bronchoarterial-coronary fistulas were also present, indicating an additional systemic arterial blood supply to the left ventricular myocardium. Transthoracic echocardiography revealed that the left ventricle was normal in size, with normal global systolic function, but with hypokinesia in the apical regions. There was no mitral valve regurgitation. Invasive coronary angiography revealed a lack of origin of the LCA from the aorta, but rather retrograde filling via collaterals from the RCA, with the contrast agent crossing to the pulmonary trunk (*Figure 2* and *Movie 1*). There was also a small, non-significant left-to-right shunt at the LCA ostium, as determined by oximetry. Blood flow from the RCA via collaterals to the anomalous LCA and on to the pulmonary trunk was confirmed. Pulmonary artery pressures were normal. Selective catheterization of the LCA through the pulmonary trunk was difficult and eventually abandoned.

**Figure 3:**
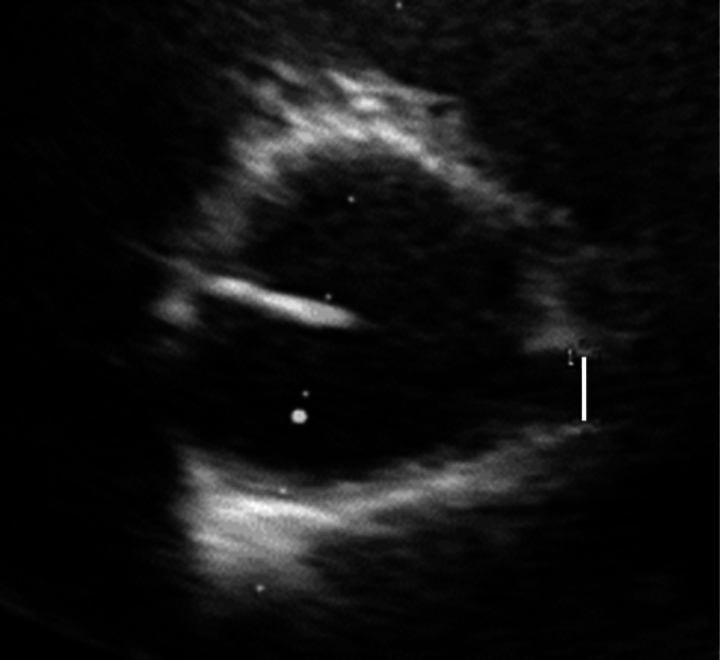
Transthoracic echocardiography. Parasternal cross-sectional view shows the dilated origin (white line) of the right coronary artery.

**Figure 4:**
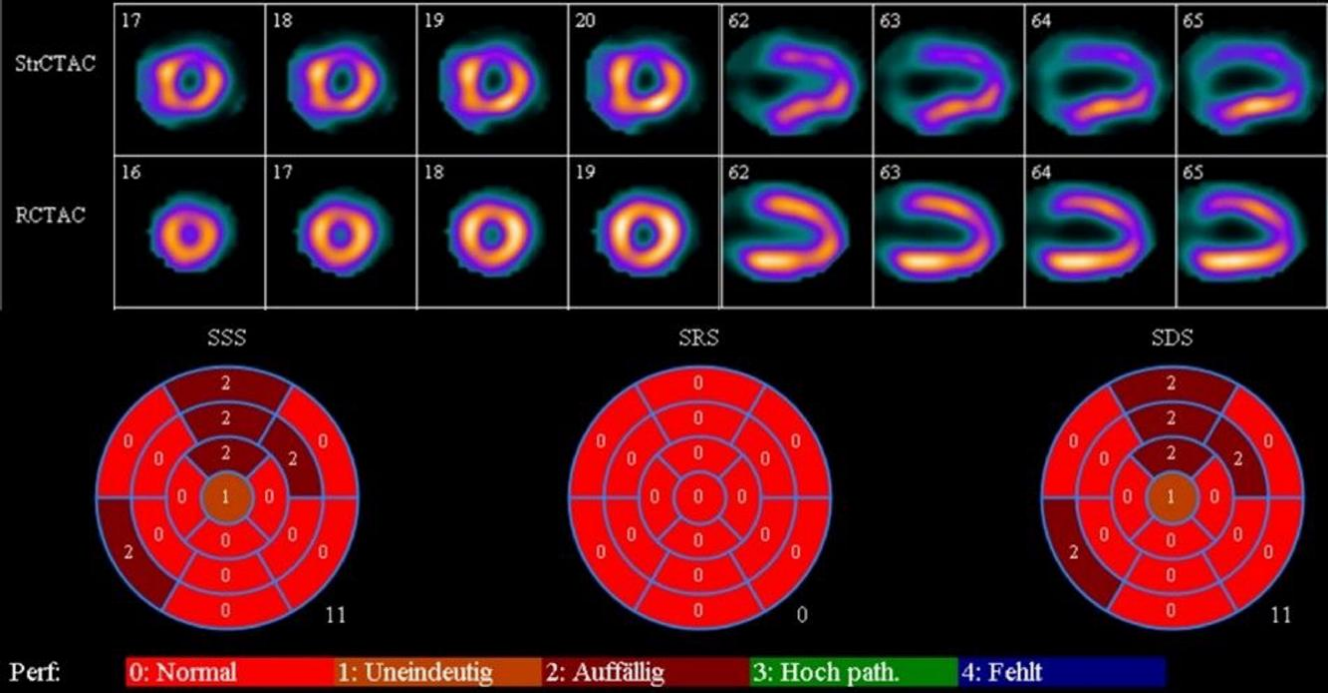
Myocardial perfusion single-photon emission computed tomography (SPECT). Under stress (StrCTAC), there is reduced uptake in the anterior wall (domain of the left coronary artery) compared to rest (RCTAC) as seen in axial (17–20/16–19) and sagittal (62–65) views. SDS is 11, which indicates that ∼16% of the myocardium is affected by reversible stress-induced ischemic changes. StrCTAC, stress conventional tomographic angular correction; RCTAC, rest conventional tomographic angular correction; SSS, summed stress score; SRS, summed rest score; SDS, summed difference score.

**Figure 5:**
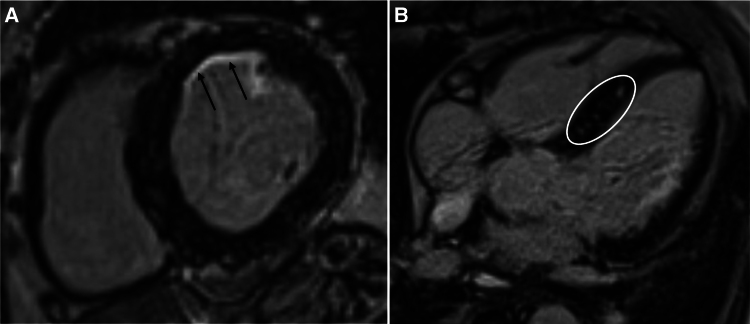
Cardiac magnetic resonance imaging (CMRI). (*A*) Late gadolinium enhancement short-axis view shows subendocardial enhancement of <50% of the anterior wall (arrows). (*B*) The four-chamber view indicates presence of prominent septal arterial collaterals (circle).

No significant arteriosclerotic coronary artery disease (CAD) was evident in CCTA (*Figure 1*) and ICA (*Figure 2*). Both (*Figures 1B* and *2*) as well as echocardiography (*Figure 3*) showed dilatation of the dominant RCA. Myocardial perfusion single-photon emission computed tomography (SPECT) (*Figure 4*) revealed a significant, reversible, stress-induced perfusion defect affecting 16% of the left ventricular myocardium (the threshold of clinical significance is ≥10%). Cardiac magnetic resonance imaging revealed small subendocardial late gadolinium enhancement (LGE) covering <50% of the anterior wall, from basal to the mid-ventricular region (*Figure 5A*), as well as prominent septal collaterals (*Figure 5B*).

Adult-type ALCAPA was diagnosed. The surgical options [if feasible, coronary reimplantation with button transfer; alternatively, aortocoronary bypass grafting with LCA origin closure; or prophylactic cardioverter-defibrillator (ICD) implantation] were discussed in detail with the patient. By the time of resubmission, the patient had not made a definitive decision; he was going to seek a second opinion in another centre. After diagnosis, he agreed to start a prophylactic beta-blocker therapy (bisoprolol 1.25 mg a day) to decrease the risk of SCD. Bisoprolol was tolerated well; however, due to bradycardia at rest, the dose could not be increased during follow-up. He stayed in continuous contact and remained asymptomatic. No imaging follow-up examinations are currently planned until his decision. The main problems he encountered in the decision-making process were the facts that he was asymptomatic throughout his whole life, despite undertaking heavy physical exercise, particularly in his younger years (e.g. serving as a soldier for several years), and that the recommendation of guidelines is major cardiac surgery.

## Discussion

Anomalous left coronary artery from the pulmonary artery earlier was typically diagnosed post-mortem by autopsy.^6^ Since the 1960s, ICA had been the leading method of diagnosis, and the first two non-invasively diagnosed cases were published in the 1990s.^6^ Coronary computed tomography angiography (CCTA) can visualize the aberrant coronary artery and collaterals.^3^ Invasive coronary angiography, the former gold standard, can also unequivocally make the diagnosis by showing retrograde LCA filling and even its aberrant origin. However, CCTA is superior in visualizing the exact anatomy.^3^ In children, in particular, echocardiography can directly show the connection of the LCA and the pulmonary artery, as well as retrograde blood flow. It can also show a hyperechogenic mitral valve papillary muscle in many cases.^3^

International guidelines recommend surgical therapy for ALCAPA, independently of the patient’s age or clinical symptoms to reduce progressive myocardial ischaemia and the risk of SCD (Grade 1C recommendation).^7,8,12^ However, of the adults reviewed by Yau *et al*., 4% of patients <50 years and 37% of patients ≥50 years were treated only medically, either because they declined surgery or due to comorbidities.^6^ There are no evidence-based recommendations for medical treatment, as only case reports exist, mostly involving the administration of ß-blockers.^12^

The presentation of our asymptomatic patient is not straightforward. Anomalous left coronary artery from the pulmonary artery was diagnosed by chance during a routine examination. Exercise stress testing and Holter monitoring did not reveal any significant ventricular arrhythmias. The patient has engaged in heavy physical exercise, particularly in his earlier life, and remains active in sports. If this congenital anomaly predisposes in this individual to malignant arrhythmias, it is likely that they would have occurred previously. The additional finding of dilated bronchial arteries supplying collateral flow to the LCA may offer protection in this case. Nevertheless, myocardial perfusion scintigraphy revealed reversible myocardial ischaemia in 16.5% of the left ventricular myocardium. However, CMRI revealed only a small subendocardial myocardial scar (<50%) in this region, which does not support prophylactic ICD implantation. The long-term complications of ICD implantation, particularly in physically active individuals (e.g. lead failure and inappropriate therapy), have to be considered. The patient had no significant coronary stenosis, which supported the decision to perform aortocoronary bypass grafting with origin closure, a technically easier procedure. There is still little evidence for the recommendation of surgical correction in all older asymptomatic ALCAPA patients,^7,9^ which makes advice and decisions difficult for individuals like ours. Thus, in real life, 37% of adults >50 years were managed medically, which is supported by the suggestion that there is lower risk of SCD in these patients.^6^

## Conclusion

We present a case report of ALCAPA in an adult patient without any symptoms throughout his whole life. ALCAPA was diagnosed incidentally in screening CCTA. Although surgery is recommended, the patient did not make a definitive decision by now.

## Lead author biography


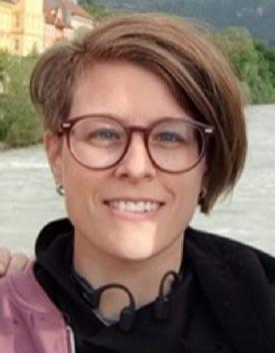


Dr Anna Jöbstl earned her medical degree from the Medical University of Vienna in 2017. From 2018 to 2024, she was a radiology resident at KH Spittal an der Drau and LKH Innsbruck. She has been working as a certified radiologist at LKH Innsbruck from January to December 2024 and at MRI-Institute Spittal an der Drau since January 2025.

## Supplementary Material

ytaf511_Supplementary_Data

## Data Availability

The data underlying this article are available in the article and in its online supplementary material.
